# Pharmacotherapy of Itch—Antihistamines and Histamine Receptors as G Protein-Coupled Receptors

**DOI:** 10.3390/ijms23126579

**Published:** 2022-06-13

**Authors:** Takemichi Fukasawa, Asako Yoshizaki-Ogawa, Atsushi Enomoto, Kiyoshi Miyagawa, Shinichi Sato, Ayumi Yoshizaki

**Affiliations:** 1 Department of Dermatology, Graduate School of Medicine, The University of Tokyo, Tokyo 113-8655, Japan; fukasawat-der@h.u-tokyo.ac.jp (T.F.); yoshizakia-der@h.u-tokyo.ac.jp (A.Y.-O.); satos-der@h.u-tokyo.ac.jp (S.S.); 2 Laboratory of Molecular Radiology, Center for Disease Biology and Integrative Medicine, Graduate School of Medicine, The University of Tokyo, Tokyo 113-8655, Japan; aenomoto@m.u-tokyo.ac.jp (A.E.); miyag-tky@umin.ac.jp (K.M.)

**Keywords:** itch, antihistamine, G protein-coupled receptors, biased agonist, inverse agonist, proactive antihistamine therapy, impaired performance, anti-PAF effect

## Abstract

Itching can decrease quality of life and exacerbate skin symptoms due to scratching. Itching not only contributes to disease progression but also triggers complications such as skin infections and eye symptoms. Therefore, controlling itching is very important in therapeutic management. In addition to the well-known histamine, IL-31, IL-4 and IL-13 have recently been reported as factors that induce itching. Itching may also be caused by factors other than these histamines. However, we do not know the extent to which these factors are involved in each disease. In addition, the degree of involvement is likely to vary among individuals. To date, antihistamines have been widely used to treat itching and are often effective, suggesting that histamine is more or less involved in itchy diseases. This review discusses the ligand-receptor perspective and describes the dynamics of G protein-coupled receptors, their role as biased agonists, their role as inverse agonists, proactive antihistamine therapy, and drug selection with consideration of impaired performance and anti-PAF effects.

## 1. Histamine as an Itch-Inducing Substance

Controlling itching is important in therapeutic management because it not only contributes to disease progression by decreasing quality of life and exacerbating skin symptoms due to scratching but also triggers complications such as skin infections and ocular symptoms. In addition to the well-known histamine, IL-31, IL-4 and IL-13 have recently been reported as factors that induce itching. It has been shown that IL-31 induces acute itch by acting on IL-31RA in sensory nerves, while IL-4 and IL-13 induce chronic itch by acting on sensory nerves and lowering the response threshold to pruritogenic factors [1]. Factors other than histamine can cause itching, but at this time we do not know the extent to which these factors are involved in each disease. Although the degree of involvement may vary from person to person, antihistamines are still widely used as treatment for itching and are often effective, suggesting that histamine is involved to a greater or lesser extent in itchy diseases. This review discusses antihistamines in terms of ligands and receptors.

## 2. Actions of Histamine on Skin and Central Nervous System

The main points of action of histamine are in the skin and brain. Because histamine has a different point of action in the skin and in the brain, histamine’s action in each is also different. In skin, histamine is primarily released from mast cells and then binds to histamine H1 receptors (“H1 receptors”) on microvascular endothelial cells. The result is itching along with wheals and erythema, such as urticaria. Histamine in the brain, on the other hand, is released from activated neurons (histamine neurons). Free histamine acts on H1 receptors in the cerebrum, cerebellum, and spinal cord as a neurotransmitter, and it induces the following major physiological functions: maintenance of arousal level, increase in spontaneous locomotion, enhancement of memory and learning functions, suppression of feeding behavior, and suppression of seizures.

## 3. Histamine-Mediated Skin Diseases

Urticaria is a skin disease in which histamine is directly involved. Atopic dermatitis, like urticaria, also presents itching, but reports on the association with histamine are conflicting. Such peripheral itching is divided into histamine-dependent and histamine-independent itching, depending on clinical sensitivity to antihistamines. Although the ratio of each in each disease has not been clarified, because histamine-dependent itching is thought to be involved in urticaria, atopic dermatitis, and eczema, in which itching is observed, antihistamines are relatively commonly used for these skin diseases in Japan.

Histamine-dependent itching is literally itching caused by histamine. It acts on the “sensory nerve” that perceives pain and itching, and the stimulus is transmitted to the brain as itching, as well as to the nerve endings, causing the release of neurotransmitters called neuropeptides. This neuropeptide then stimulates mast cells to secrete histamine and other substances. Stimuli such as “itching and scratching” stimulate the sensory nerves in sensitive skin, causing the release of neuropeptides, which in turn stimulate the secretion of the itching substance histamine. It is believed that this phenomenon causes the itching to spread more and more.

In itchy skin, inflammation is often present, and several inflammatory cytokines have been reported to bind directly to receptors on neurons and trigger itch signals. IL-31 is produced primarily by Th2 cells [2]. Receptors for IL-31 are also expressed on neurons and are thought to be mediators that play a central role in the itching that occurs especially in patients with atopic dermatitis and pruritus.

Receptors for IL-4 and IL-13, also cytokines produced by Th2 cells, are also expressed on nerve endings and directly cause itching [1]. IL-31 has been implicated in the acute phase of inflammation, while IL-4 and IL-13 are involved in the chronic phase of inflammatory itching. Suppression of IL-4 and IL-13 directly affects not only the suppression of inflammation, but also the suppression of itching.

In addition, IL-17, which plays an important role in the pathogenesis of psoriasis, is also known to be a mediator that can directly activate the itch nerve [3]. Thymic stromal lymphopoietin (TSLP), produced by epidermal keratinocytes, is another known mediator of itch [4]. PAR2, a receptor on the plasma membrane that recognizes proteases and is expressed on epidermal keratinocytes and neurons, is also implicated in itching. Various foreign antigens such as pollen and mite antigens have protease activity, and when PAR2 is activated, Th2-type inflammation is induced. Substance P is a neuropeptide secreted from nerve terminals. Activation of histamine receptors and PAR2 on neurons results in secondary secretion of Substance P from neurons. The receptor for Substance P (NK1-R) is expressed on epidermal keratinocytes, vascular endothelium, mast cells, and even neurons, and it is known to amplify dermatitis and itching. Therefore, it is thought to act as an enhancer rather than an initiator of itch signals.

MRGPR is associated with histamine-independent itching in atopic dermatitis and other conditions. MRGPR is a receptor on the plasma membrane and is selectively expressed on mast cells and neurons. Chloroquine is known as a ligand, but the details of the endogenous ligand are unknown. Mast cells express both IgE receptors and MRGPRs, but the nature of the itch caused when each is activated is different [5]. When IgE receptors are activated, histamine is secreted by mast cells, causing a fast-rising, short-duration itch. On the other hand, when MRGPRB2 on mast cells is activated, tryptase and other enzymes are secreted, which in turn activate MRGPRA3 and MRGPRD on neurons, causing itching. Itching at this time is considered to be a slow-rising, persistent itch. Thus, itching is caused by various substances other than histamine, and histamine and its receptors are discussed below.

## 4. Regulation of the Function and Distribution of Histamine Receptors as G Protein-Coupled Receptors—Desensitization Therapy—

**Figure 1:**
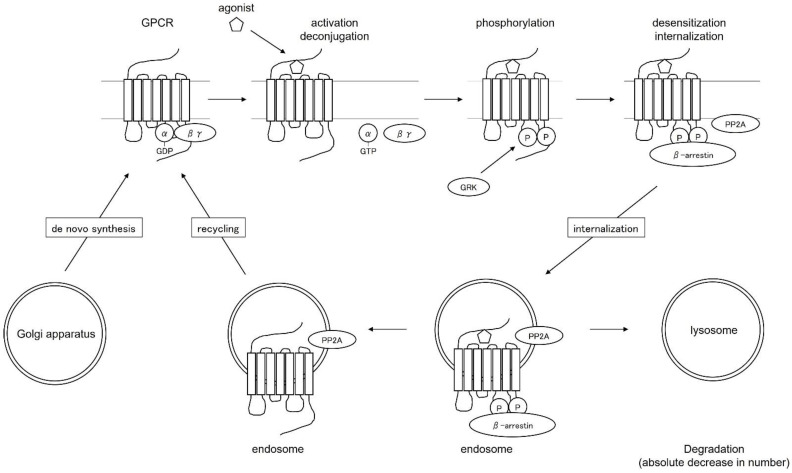
Regulation of function and distribution of G-protein-coupled receptors.

The H1 receptor is a Gq protein-coupled receptor with a seven-transmembrane structure. G protein-coupled receptors (GPCRs) are activated when stimulated by agonists, which activate G proteins for signaling and at the same time serve as substrates for G-protein-coupled receptor kinase (GRK), which is phosphorylated. The phosphorylated receptor binds to β-arrestin and the receptor and G proteins are no longer able to bind (deconjugation). β-arrestins are coated with clathrin and transport receptors into the cell. Receptors that have migrated into the cell are resensitized and recycled to the plasma membrane as normal receptors. Some of the receptors transported into the cell are degraded by lysosomes, resulting in a decrease in the absolute number of receptors (Figure 1). The function and distribution of GPCRs are regulated by these mechanisms.

There are three major types of changes in the number and function of H1 receptors on the cell surface: changes in “absolute number” due to the balance between new synthesis and degradation, changes in “distribution” due to intracellular translocation and recycling to the plasma membrane, and changes in “function” due to changes in receptor ligand affinity and deconjugation with G proteins. Functional changes are caused by changes in receptor ligand affinity and deconjugation with G proteins. This mechanism of desensitization has been applied to drugs for multiple sclerosis and LH-RH-like drugs. It has been reported that H1 receptor expression is elevated in diseases associated with itching, such as atopic dermatitis and urticaria [6], suggesting the possibility of therapeutic application to these diseases.

## 5. Intracellular Signaling Mechanism via GPCRs

While there are more than 800 GPCRs in the genome, only seven kinases (GRK1 to GRK7) phosphorylate GPCRs and act as triggers for desensitization. GRK1 to GRK7) [7]. Three of these (GRK1/4/7) are expressed only in a very limited number of tissues, suggesting that four of them (GRK2/3/5/6) mediate desensitization in many tissues. The mechanism of desensitization was proposed by analysis using GRK2. Three types of GRK2/5/6 are thought to be involved in signals that are not mediated by G proteins. 

β-arrestins belong to the arrestin family, which has four subfamilies [8]. There are four types of arrestins: two types of arrestins and beta-arrestins (beta-arrestin 1 and beta-arrestin 2) that regulate photoreception and are expressed in the rods or cones of the retina. Expression of β-arrestins is found in many tissues. β-arrestin 1 and β-arrestin 2 act as intermediary molecules for GPCR desensitization and G protein-independent signaling. Some GPCRs may show beta-arrestin selectivity for desensitization.

The most advanced analysis of signaling through GRKs is GRK2. GRK2 expression is high in cardiac myocytes, and its expression and activity are further increased upon exposure to stress, such as during myocardial infarction, which has been analyzed in detail in relation to cardiac disease [9]. The results revealed that GRK2 acts as one of the kinases that induce stress-induced cell death in the heart. The carboxyl-terminal domain of GRK2 (GRK2-ct, formerly called βARK-ct) is used for functional analysis of GRK2 [10]. The carboxyl terminus of GRK2 contains a binding site for the βγ subunit of G protein, and binding of the βγ subunit to this domain is essential for GRK2 activation. The βγ subunit released from the G protein facilitates the translocation of GRK2 to the plasma membrane and increases its kinase activity. GRK2-ct inhibits this transition and activation, thereby blocking GRK2-mediated responses. The carboxyl terminus of GRK2 contains a phosphorylation site of extracellular signal-regulated kinase (ERK), and phosphorylation by ERK is reported to be essential for mitochondrial entry and induction of cell death [11]. Because GRK2-ct contains a phosphorylation site by ERK, it also inhibits ERK phosphorylation and suppresses mitochondrial entry and induction of cell death. The mechanism by which GRK2 induces cell death in mitochondria is not clear. GRK2 actions in cells other than cardiomyocytes have also been reported. It has been reported to directly interact with Akt kinase and inhibit its activity, to participate in cell migration via interaction with GPCR-kinase interactingprotein1 (GIT1), and to enhance GPCR internalization by promoting membrane migration of phosphoinositide3-kinase (PI3K). It has also been shown to phosphorylate insulin receptor substrate 1 (IRS-1) and suppress signaling through the insulin receptor, and to be involved in Smad signaling and nuclear factor kappa-B (NF-κB) signaling. Thus, the role of GRK2 as a molecule that mediates cellular responses is being established not only in the heart but also in many other tissues. 

It has been reported that GRK2 and GRK6, two of the seven GRKs, are involved in phagocytosis of apoptotic cells [12]. GRK6 mediated phagocytosis through a novel pathway dependent on kinase activity and independent of previously known pathways. There are several reports on the involvement of GRK5 in signaling. GRK5 suppresses mTOR (mammalian target of rapamycin) signaling through its interaction with Raptor (regulatory-associated protein of mTOR), a component of mTORC1. GRK5 is known to be an inhibitor of apoptosis induced by DNA damage through phosphorylation of p53. GRK5 acts as a histone deacetylase (HDAC) kinase in cardiac myocytes and is known to mediate cardiac hypertrophic responses [13].

The fact that β-arrestin mediates signaling was discovered in the process of analyzing the activation of ERK by GPCR. In other words, β-arrestin recruits sarcoma kinase (Src) to the β-adrenergic receptor, and inhibition of binding to this Src kinase inhibits ERK activation [14]. β-arrestin acted as an essential protein for ERK activation. 

## 6. Antihistamines as Biased Agonists

**Figure 2:**
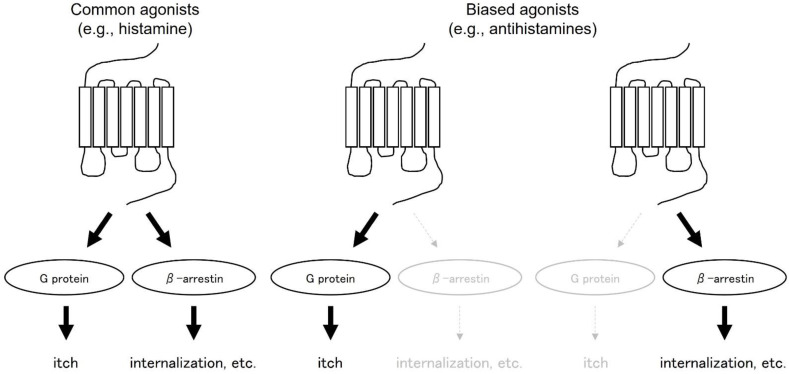
Biased agonist.

In addition to mediating desensitization, β-arrestins are actively involved in signal transduction as scaffold proteins. The β-arrestin pathway is separate and distinct from the G-protein pathway. Agonists that activate either the G protein or the β-arrestin pathway are called biased agonists (Figure 2). Common physiological agonists, such as histamine, activate G proteins, causing itching, as well as beta-arrestins, causing receptor internalization, etc. Therefore, it has both functions. On the other hand, some biased agonists, such as antihistamines, are more effective in suppressing G-protein-only signals that cause itching, while others are more effective in suppressing beta-arrestins that cause receptor internalization, etc. In a paper examining the function of antihistamines as biased agonists on H1 receptor desensitization, internalization, G-protein-independent pathways, and transcriptional regulation of inflammatory cytokines [15], three antihistamines, diphenhydramine, triprolidine, and chlorpheniramine, are examined. Pretreatment with triprolidine and chlorpheniramine desensitized H1 receptors, while diphenhydramine and triprolidine caused internalization. It becomes clear that desensitization and internalization of the receptors by these antihistamines occurs by a different mechanism than phosphorylation by GRK2. The reduced number of cell surface receptors was found to be restored by de novo synthesis, and the increase in COX-2 and IL-8 mRNA was suppressed after long-term exposure to antihistamines, followed by histamine exposure after removal of the antihistamines. The above revealed the properties of antihistamines as biased agonists.

## 7. Antihistamines as Inverse Agonists

**Figure 3:**
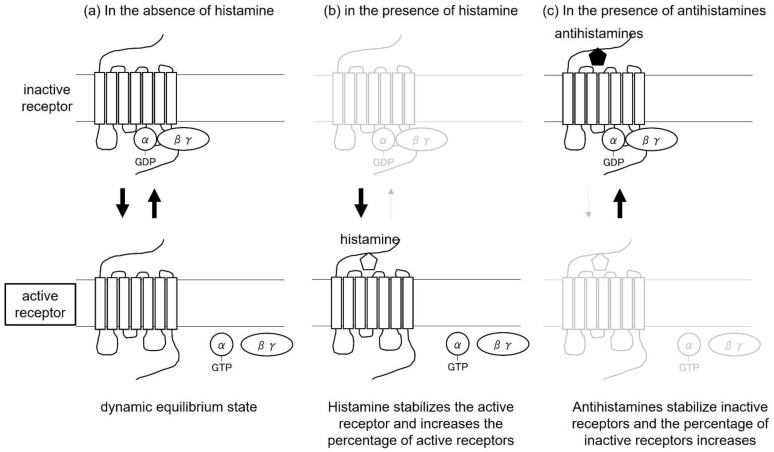
Inverse agonist concept. (**a**) In the absence of histamine, inactive and active receptors are in dynamic equilibrium. (**b**) In the presence of histamine, histamine stabilizes active receptors and increases the percentage of active receptors. (**c**) In the presence of antihistamines, antihistamines stabilize inactive receptors and increase the proportion of inactive receptors.

Apart from biased agonists, antihistamines have been thought to produce therapeutic effects by acting as antagonists that block histamine binding to the H1 receptor. However, it has recently been shown that H1 receptors have active receptors that are activated even in the absence of histamine, and that the two types of receptors, active and inactive, maintain a dynamic equilibrium [16]. Histamine acts on the active receptor, shifting the equilibrium in the direction of increasing the percentage of active receptors. On the other hand, antihistamines act on inactive receptors and exert their effects in the direction of increasing the percentage of inactive receptors and decreasing the percentage of active receptors. Under the active receptor, the G protein is activated and the signal is transmitted into the cell. This induces itching and internalization of β-arrestin-mediated receptors into the cells. Under inactive receptors, antihistamines are more effective because G proteins are not activated and internalization of receptors via β-arrestins is thought to be less likely to occur. This action of antihistamines to stabilize inactive H1 receptors in the absence of histamine is called inverse agonist (Figure 3).

## 8. Relationship between Biased Agonists and Inverse Agonists

β-arrestin-mediated biased agonists are classified as antagonists because they activate only β-arrestins and not G proteins. Antagonists are classified into two types: inverse agonists, which reduce basal activity, and neutral antagonists, which do not affect basal activity. Inverse agonists reduce basal activity, while biased agonists are a distinct concept because of the β-arrestin-mediated pathway.

## 9. Significance in Therapy—Inverse Agonists, Desensitization Therapy, and Biased Agonists—

Changes in the conformation of H1 receptors on the plasma membrane and their distribution by intracellular transfer are involved in the progression of desensitization. In addition to acting as “inverse agonists” through long-term administration of receptor antagonists, antihistamines as “desensitizers” that reduce receptor sensitivity and number, and as “bias-type agonists”, may be one allergy treatment strategy. Treatment based on these mechanisms includes prophylactic administration of antihistamines for chronic urticaria. When antihistamines are used to treat chronic urticaria and discontinued soon after symptoms improve, flare-ups often occur. Therefore, many dermatologists have empirically continued antihistamines for 1–2 months after symptoms have resolved, and for about 6 months to a year in patients with longer disease duration. In recent years, evidence has also shown that prophylactic administration of nonsedating antihistamines maintains symptom improvement and prevents recurrence [17], and the Chronic Urticaria Practice Guidelines 2018 also recommended oral antihistamines for a while after resolution or lightening of symptoms [18]. One possible reason for this evidence is that antihistamines continued to reduce the percentage of active receptors in a sustained manner.

Here are data on prophylactic administration of antihistamines for chronic urticaria. Subjects were patients with symptomatic but not bothersome improvement of chronic reed measles after 1 to 4 weeks of oral treatment with epinastine hydrochloride, a second-generation antihistamine. These patients were divided into a prophylactic group that received epinastine hydrochloride continuously and a symptomatic group that received it only when symptoms occurred, and were studied for 8 weeks. In addition, QOL assessment using Skindex-16 showed no significant improvement in any of the items in the symptomatic group, whereas the prophylactic group showed significant improvement in the symptomatic and emotional items after 8 weeks [19]. 

In another report, a second-generation antihistamine, epinastine hydrochloride, was administered prophylactically to patients with daily urticarial symptoms for 4, 8, or 12 weeks, with or without symptoms. The differences in the course of symptoms during the subsequent 12-week symptomatic treatment period are examined in terms of symptom behavior (itching, rash, etc.) and patient quality of life based on the patients’ diaries. The results showed that patients who received prophylactic epinastine hydrochloride for 12 consecutive weeks had better recurrence and maintenance of improvement rates than those who received prophylactic epinastine for only 4 weeks. Quality of life was also assessed using the Skindex-16, but no significant difference was found [17]. These reports suggest that long-term prophylactic oral administration of H1-receptor antagonists (2–3 months) in the treatment of chronic urticaria may be effective in preventing recurrence of urticaria.

The usefulness of prophylactic medication for hay fever has also been demonstrated. The study compared the efficacy of olopatadine hydrochloride, a second-generation antihistamine, over an 8-week period in patients with mild disease at the time of treatment initiation, in an initial treatment group in which treatment was started up to 7 days after the first day of pollen dispersal, and in a post-onset treatment group in which treatment was started after the first day of dispersal. According to the results, when pollen dispersal counts were less than 2000 particles/cm^2^ (low to moderate dispersal), sneezing was better in the initial treatment group than in the post-onset treatment group at all time points up to 8 weeks after the start of dispersal. In the case of 2001 to 5000 sneezes/cm^2^ (large amounts), the initial treatment group was superior up to 3 weeks and 5 weeks after the start of scattering, and in the case of 5001 or more sneezes/cm^2^ (very large amounts), the initial treatment group was superior up to 3 weeks after the start of scattering. The total score of nasal symptoms (TSS), which is the sum of the scores of the three main nasal symptoms such as sneezing attacks, nasal discharge, and nasal obstruction, showed that the scores of the initial treatment group decreased more than those of the post-onset treatment group at all time points up to 8 weeks after the start of scattering in the case of low- to moderate-dose scatter, up to 6 weeks after the start of scattering in the case of large-dose scatter, and up to 4 weeks after the start of scattering in the case of very large-dose scatter [20].

In another report, a double-blind, crossover, comparative study was conducted in which subjects with hay fever received either fexofenadine HCl or placebo for 1 week followed by intranasal antigen exposure, and after a 1-week washout period, fexofenadine HCl and placebo were swapped and administered intranasally for another week, followed by intranasal antigen exposure. According to the results of this study, subjective symptoms such as sneezing, nasal discharge, nasal congestion, itching, and watery eyes were significantly improved in the group that received oral fexofenadine hydrochloride [21].

The results for atopic dermatitis were similar to those for chronic urticaria, with 67.8% of patients in the continuous treatment group who received continuous antihistamines in addition to topical treatment not experiencing recurrence of itching during the 12-week observation period. On the other hand, 55.9% of patients in the intermittent group who received only topical treatment and a VAS (Visual Analogue Scale) score of 30 or higher for itching were treated with antihistamines, indicating a significantly higher percentage in the continuous group (*p* < 0.0001, χ^2^-test) [22]. Based on the above, prophylactic administration of antihistamines, so-called proactive therapy, may be effective in itchy skin diseases. When considering the efficacy of this proactive therapy, it is important to consider the improvement in itching and H1 receptor status separately. Even if the itching has improved, some of the H1 receptors remain and continue to be activated, which may lead to relapse of symptoms, so the treatment strategy is to administer antihistamines on an ongoing basis to control relapse.

The timing of antihistamine discontinuation should be considered based on the individual patient’s situation, including the duration of illness. In general, patients with longer disease duration require long-term use, but there is no definite time for discontinuation. Therefore, it is important to discontinue dosing while monitoring the patient’s condition, and if the patient relapses after discontinuation, it is important to restart dosing through trial and error. In order to maintain the effect, more than determining the appropriate duration of antihistamine use, more sustained relapse inhibition can be expected if the medication is continued after the itching has improved, in compliance with the dosage and administration.

## 10. Drug Selection Based on Impaired Performance

**Figure 4:**
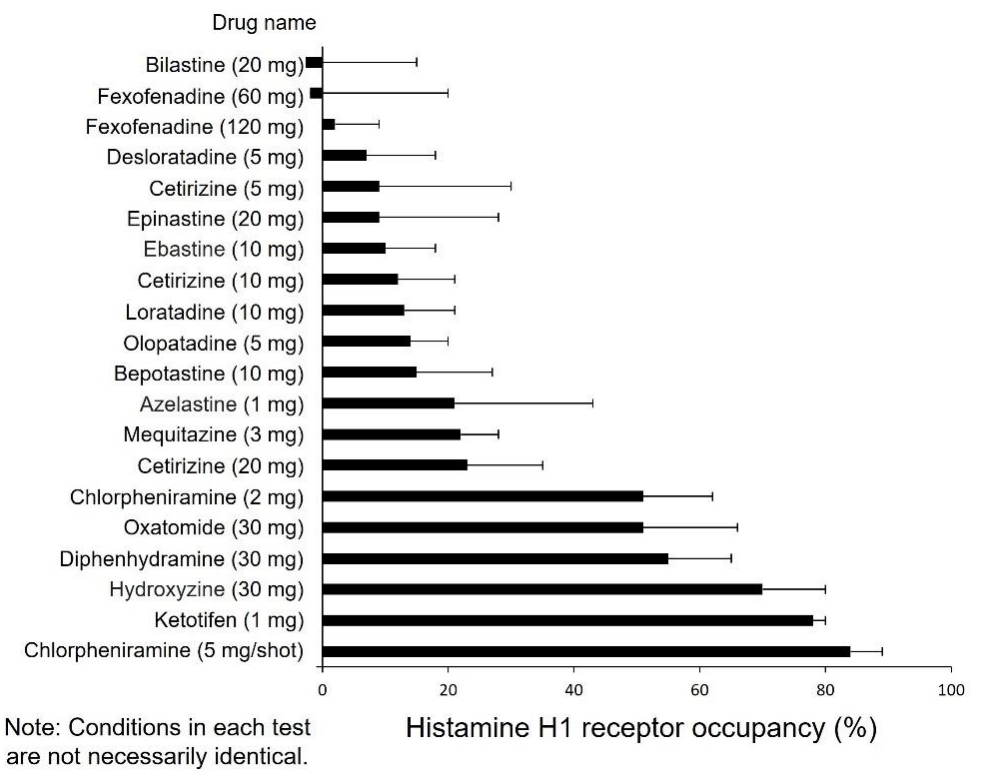
H1 receptor occupancy and sedation of antihistamines in the brain.

Another topic for antihistamine selection is impaired performance. Impaired performance is caused by the sedative effects of antihistamines, and this sedative effect is classified as sedative when the H1 receptor occupancy in the brain is 50% or more, mildly sedative when it is 50–20%, and nonsedative when it is 20% or less, with first-generation antihistamines being sedative and second-generation antihistamines being non-sedative (Figure 4) [23,24]. This brain occupancy is often understood as the binding rate of histamine to the H1 receptor, but it is thought to reflect the blood–brain barrier passability because the brain occupancy of the H1 receptor is proportional to the strength of the sedative effect [25]. The functional group of the drug is involved in the blood–brain barrier passage, and it is thought that second-generation antihistamines are less likely to cross the blood–brain barrier and less likely to have impaired performance because their hydrophilic carboxyl or amino groups are replaced, making them more easily ionized under physiological conditions. Therefore, it is considered important to select a second-generation antihistamine that makes it as difficult to cross the blood–brain barrier as possible when using proactive therapy with the antihistamines we discussed earlier, because the treatment is relatively long-term, and while it has been shown to have a sustained effect on itching, it should have less concern about side effects such as drowsiness. It should be noted, however, that each drug may be a biased agonist, an inverse agonist, or a neutral antagonist. This is because each drug has a stronger inhibitory effect on G protein activation or a stronger effect on receptor internalization. Another approach is to suppress active receptors and increase the percentage of inactive receptors. In actual clinical practice, there are many cases in which brain H1 receptor occupancy does not necessarily correlate with sedation. Probably, this is due in part to the fact that each patient’s ability to metabolize drugs and the kinetics of drugs varies. Therefore, there is a need to actually use the product on the patient and consult with the patient carefully to determine how the sedative properties are and whether it is appropriate for the patient in front of the eyes. Proactive therapy should then be conducted after evaluation in individual patients to determine the degree of impaired performance.

## 11. Drugs with a New Mechanism of Action; Anti-PAF Action

**Figure 5:**
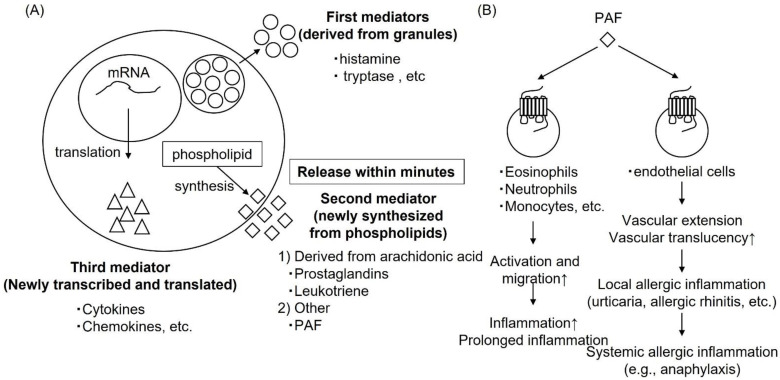
Mechanisms by which mediators released from mast cells (**A**) and PAF induce allergic inflammation (**B**). (**A**) Circles mean first mediators derived from granules, such as histamine or tryptase. Squares mean second mediators newly synthesized from phospholipids, such as prostaglandins, leukotrienes, which derived from arachidonic acid, or PAFs. Triangles mean third mediators newly transcribed and translated, such as cytokines or chemokines.

Recently, in addition to the H1 receptor inhibitory effects of conventional antihistamines, some drugs have also shown platelet-activating factor (PAF) receptor inhibitory effects [26,27] (Figure 5). There are three main types of mediators released by cells, such as mast cells, for example. The primary mediators are histamine and tryptase, which are derived from intracellular granules. Secondary mediators include prostaglandins and leukotrienes derived from arachidonic acid, which are newly synthesized from phospholipids, as well as PAF. The third mediators, newly transcribed and translated, include cytokines and chemokines [28,29,30,31,32,33,34]. They differ in the time lapse between stimulation and release from the cell. The first mediator is quick, within minutes, while the third takes several hours (Figure 5A). PAFs are produced by a variety of cells, including mast cells, vascular endothelial cells, epithelial cells, platelets, neutrophils, eosinophils, and monocytes. Furthermore, these PAF-producing cells also express the PAF receptor, which amplifies the PAF response by autocrine. PAF is involved in the appearance of urticaria and acts on vascular endothelial cells, increasing vascular permeability and causing wheals. PAF acts on PAF receptors on the plasma membrane of eosinophils, neutrophils, and monocytes, causing cell activation and migration, leading to increased and prolonged inflammation. It also acts on PAF receptors on the cell membrane of vascular endothelial cells, causing vasodilatation and vascular permeability, leading to local allergic inflammation such as urticaria and allergic rhinitis [35,36,37,38]. Severe cases cause anaphylaxis and other allergic inflammation throughout the body (Figure 5B). It also activates mast cells via peripheral nerve activation, which promotes degranulation and histamine release. The addition of anti-PAF action to the H1 receptor inhibitory action may reduce itching more effectively. Because PAF receptors, like H1 receptors, are G-protein-coupled receptors, prophylactic administration of antihistamines, so-called proactive therapy, may be equally effective.

## 12. Conclusions

Antihistamines as desensitizers, inverse agonists, and biased agonists may be effective for prophylactic administration and prevention of recurrence in a variety of allergic diseases with itching. The use of antihistamines as such proactive therapy should be based on the concept of desensitization therapy, inverse agonists, and biased agonists. It would then be advisable to select antihistamines with an awareness of their anti-PAF action as a new mechanism of action, while considering those with less impaired performance.

## Data Availability

Not applicable.
